# Obstructive Sleep Apnea Patients Having Surgery Are Less Associated with Glaucoma

**DOI:** 10.1155/2014/838912

**Published:** 2014-07-24

**Authors:** Hsin-Yi Chen, Yue-Cune Chang, Che-Chen Lin, Fung-Chang Sung, Wen-Chi Chen

**Affiliations:** ^1^Graduate Institute of Clinical Medical Science, China Medical University, Taichung 404, Taiwan; ^2^Department of Ophthalmology, China Medical University Hospital, Taichung 404, Taiwan; ^3^Department of Mathematics, Tamkang University, New Taipei City 25137, Taiwan; ^4^Management Office for Health Data, China Medical University Hospital, Taichung 404, Taiwan; ^5^Department of Public Health, China Medical University, Taichung 404, Taiwan; ^6^Department of Urology, China Medical University Hospital, Taichung 404, Taiwan

## Abstract

*Objective.* To investigate if different treatment strategy of obstructive sleep apnea (OSA) was associated glaucoma risk in Taiwanese population.* Methods.* Population-based retrospective cohort study was conducted using data sourced from the Longitudinal Health Insurance Database 2000. We included 2528 OSA patients and randomly selected and matched 10112 subjects without OSA as the control cohort. The risk of glaucoma in OSA patients was investigated based on the managements of OSA (without treatment, with surgery, with continuous positive airway pressure (CPAP) treatment, and with multiple modalities). The multivariable Cox regression was used to estimate hazard ratio (HR) after adjusting for sex, age, hypertension, diabetes, hyperlipidemia, and coronary artery disease.* Results.* The adjusted HR of glaucoma for OSA patients was 1.88 (95% CI: 1.46–2.42), compared with controls. For patients without treatment, the adjusted HR was 2.15 (95% CI: 1.60–2.88). For patients with treatments, the adjusted HRs of glaucoma were not significantly different from controls, except for those with CPAP (adjusted HR = 1.65, 95% CI = 1.09–2.49).* Conclusions.* OSA is associated with an increased risk of glaucoma. However, surgery reduces slightly the glaucoma hazard for OSA patients.

## 1. Introduction

Obstructive sleep apnea (OSA) is characterized by total or partial obstruction of the upper airway, which leads to impaired sleep, autonomic dysfunction, and transient nighttime hypoxemia [1, 2]. Several ocular disorders related to OSA were reported [3]; among them, glaucoma has been shown to have strong relationship with OSA [4–6]. However, reports on the association between OSA and glaucomatous optic neuropathy may show conflicting results [7, 8]. In Lin et al.'s study, OSA is associated with an increased risk of open angle glaucoma (OAG) diagnosis during a 5-year follow-up period [4]. In other Lin et al. [5] and Sergi et al.'s [6] studies, they both reported strong association between normal tension glaucoma (NTG) and OSA [5, 6]. But, Girkin et al. [9] and Stein et al. [10] failed to find a positive association between OSA and glaucoma. To better understand the association between these two conditions in Taiwanese population, we studied a population-based dataset from the Taiwan National Health Insurance Program. We also evaluated if different management methods would influence the risk of glaucoma in OSA.

## 2. Patients and Methods

### 2.1. Data Source

The Taiwan National Health Insurance was a nationwide, single-payer insurance established in 1996 and covered 99% Taiwan citizen since 1998. The National Health Research Institutes involved all reimbursement claim data to establish and maintain the National Health Insurance Research Database. All personal information was encoded to protect personal privacy with surrogated identification before being released to the research.

The study used the Longitudinal Health Insurance Database which was a subset of National Health Insurance Research Database. The Longitudinal Health Insurance Database randomly selected one million individuals and was constructed of annual claim data. This claim dataset provided scramble and anonymous identification numbers to connect to the each person's relevant claim information, including the individual's sex and date of birth, registry of medical services, and medication prescriptions.

All of the individual's disease history was recorded from inpatient and outpatient files and disease registry based on the International Classification of Diseases, Ninth Revision, Clinical Modification (ICD-9-CM).

### 2.2. Study Population

This research was a population-based retrospective cohort study. The OSA cohort collected the newly diagnosed OSA (ICD-9-CM 780.51, 780.53, and 780.57, and with the polysomnography exam) patient in 2000–2009. Polysomnography, consisting of a simultaneous recording of multiple physiologic parameters related to sleep and wakefulness, is often considered the criterion standard for diagnosing OSA, determining the severity of the disease, and evaluating various other sleep disorders that can exist with or without OSA [11]. The index date was set on the OSA diagnosed day. The control cohort was selected from the individuals never with OSA diagnosis in Longitudinal Health Insurance Database and 4-fold frequency matched by age (per 5 years) and sex and randomly assigned the date with the same index year of the OSA cohort as index date. Both of these two cohorts excluded the individual with the glaucoma diagnosed before index date. The major event of the research was glaucoma diagnosed based on ICD-9-CM 365 (365.1, 365.2, and 365.9). The follow-up was ended when the individual had glaucoma (defined as event time) or withdrawn the insurance or until December 31, 2010 (defined as censoring time).

We also investigated the effect of OSA treatment to the risk of glaucoma. The OSA patients were divided into 4 groups: (1) without any treatment, (2) with surgery (pharyngeal or nasal surgery), (3) with continuous positive airway pressure (CPAP), and (4) with multiple modalities (both surgery and CPAP). The types of pharyngeal surgery included were tonsillectomy (T), adenoidectomy (A), adenotonsillectomy (T&A), uvulopalatopharyngoplasty (UPPP), and laser assisted uvuloplasty (LAUP). The nasal surgery contained septoplasty, turbinoplasty, stomatoplasty, and laser turbinoplasty.

We considered the following comorbidities as possible confounding factors. The comorbidities included hypertension (ICD-9-CM 401–405), diabetes (ICD-9-CM 250), hyperlipidemia (ICD-9-CM 272), and coronary artery disease (CAD, ICD-9-CM 410–414) from inpatient and outpatient file before index date.

### 2.3. Statistical Analysis

The distributions of these two cohorts were showed by mean and standard deviation (SD) for age and number and proportion for sex and comorbidities. The* t*-test and chi-square test were applied to test the difference of continuous variable and category variables between comparison cohort and OSA cohort, respectively. We calculated the cumulative incidence rate as the number of new occurrence cases divided by the contributed person years for each individual in the cohort. The curves of cumulative incidence rates of glaucoma were estimated by Kaplan-Meier method. To adjust for the confounding variables' effects, the stratified Cox proportional hazard models (or Cox regression) were used to compare the relative risks (in terms of the hazard rates ratios, HRs) between comparison cohort and OSA cohort with different managements.

All data managements and statistical analyses used SAS v.9.3 (SAS Institute, Cary, NC, USA) statistical package. The curves of cumulative incidence rates were drawn by R software (R Foundation for Statistical Computing, Vienna, Austria). Statistical significance was defined as *P* < 0.05.

## 3. Results

There were 2528 individuals with OSA and 10112 individuals without OSA (comparison cohort). In Table 1, these two cohorts had the same mean age (45.1 years) and sex ratio (77.8% for male). In the OSA cohort, 1431 individuals (56%) never received any treatment, 24% OSA individual received CPAP treatment only, 11% OSA individuals had surgery only, and others had multiple modality. The proportion of individuals without any comorbidity in the OSA cohort was significantly less than that of the comparison cohort (50.0% versus 71.9%, *P* value < 0.0001). As shown in Table 1, the proportions of comorbidities of hypertension, diabetes, hyperlipidemia, and coronary artery disease in the OSA cohort were significantly greater than those in the comparison cohort, respectively (all *P* value < 0.0001).

**Figure 1:**
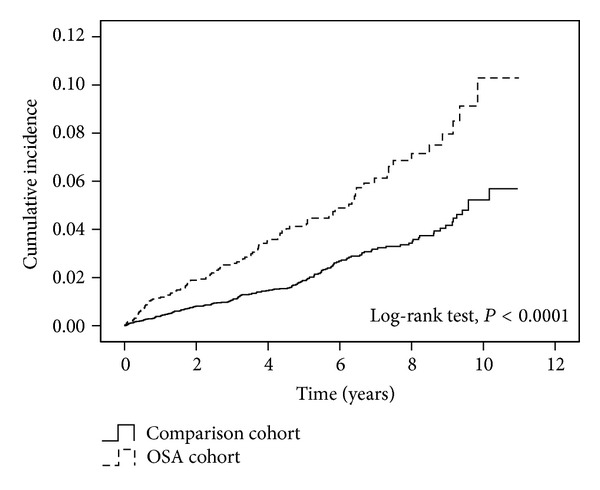
Cumulative incidence rates of glaucoma in study population.

Table 2 demonstrates the incidence rates of glaucoma and the estimated rates ratios between two study cohorts. The total incidence rates of glaucoma were 90.91 and 42.89 per 10000 person-years in the OSA cohort and the comparison cohort, respectively. In Figure 1, the result of log-rank test showed that the cumulative incidence curve for the OSA cohort was significantly higher than that of the comparison cohort (*P* < 0.0001). After adjusting for the effects of listed confounding factors in Table 2, compared to the comparison cohort, the OSA cohort had a significant 1.88-fold instantaneous occurrence rate of glaucoma (HR = 1.88, 95% CI for HR = 1.46–2.42).

The results of stratified analyses based on age group, sex, and comorbidity for these two cohorts were also showed in Table 2. The adjusted hazard rates ratios of glaucoma for OSA versus comparison cohort in age groups, divided as less than 44 years, 45 to 54 years, and higher than 55 years, were 2.42, 1.64, and 1.65, respectively. Similarly, the adjusted hazard ratios of glaucoma for OSA versus comparison cohort in female and male were 2.23 and 1.75, respectively, with corresponding 95% CI, 1.37–3.65 and 1.30–2.35. In the individual without any comorbidities, the adjusted risk of glaucoma was 2.40 in OSA cohort (95% CI = 1.58–3.66).

**Figure 2:**
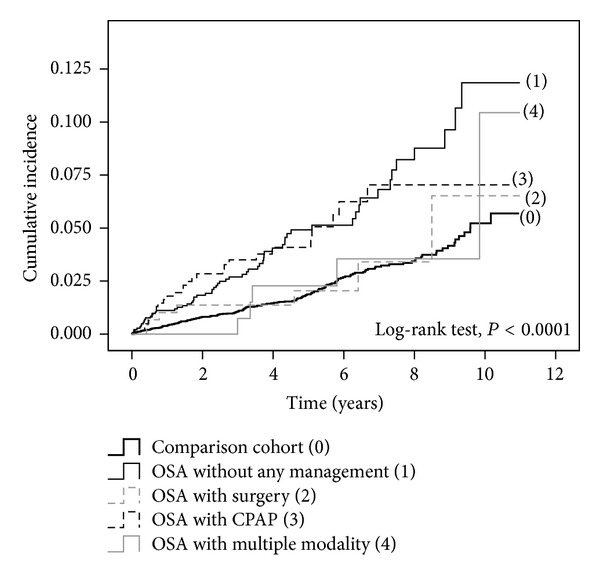
Cumulative incidence rates of glaucoma in study with/without management.

The incidence rates of glaucoma in comparison cohort and OSA cohort with/without managements were shown in Table 3. The results of log-rank test for the equality of the cumulative incidence rates among OSA patients with different managements were presented in Figure 2 (*P* < 0.0001). To compare the relative risks of glaucoma among the aforementioned groups, the stratified Cox regression model was used to adjust for the effects of age, sex, hypertension, diabetes, CAD, and hyperlipidemia simultaneously. As shown in Table 3, the relative risk (HR) for those OSA patients without any treatment versus comparison cohort was 2.15 (95% CI, 1.60–2.88). For those OSA with treatment, the relative risks of glaucoma were not significantly different from the comparison cohort except the group with CPAP treatment only (HR = 1.65, 95% CI = 1.09–2.49).

## 4. Discussion and Conclusion

### 4.1. Discussion. 

This study used population-based data to examine the association between glaucoma and OSA in Taiwan. Common comorbidities possibly related to both glaucoma and OSA, including hypertension, diabetes, hyperlipidemia, and coronary artery disease, were evaluated. The result shows that OSA patients have significantly higher risks of all considered comorbidities than the comparison group. To adjust the effect of age, sex, and all comorbidities, the relative risk of glaucoma for OSA versus comparison cohort was 1.88 (adjusted HR = 1.88, 95% CI: 1.46–2.42). The exact relationship between OSA and glaucoma remains unclear, with smaller prospective studies reporting a positive association but larger retrospective cohort studies declaring no association [7, 8]. Here we report that OSA is associated with an increased risk of glaucoma based on a large claim database. Although this work again confirms the relationship between OSA and glaucoma, some interesting points should be mentioned. First, it is difficult to directly compare our study with others because of different study design and method. For example, in our study, both OSA and glaucoma cases were defined on the basis of ICD-9 coding and therefore subjected to miscoding and possible underdiagnosis or misdiagnosis. And the definition of OSA among studies was quite different. Not all studies apply PSG testing as diagnostic criteria for OSA. Some used questionnaire or symptom/sign only, which might limit its validity. Second, we did not limit our samples to OAG only; it is not proper to directly compare ours to those studies evaluating OAG or NTG only. In one recent study by Park et al. [12], including different types of glaucoma (primary OAG, chronic angle closure glaucoma, pigmentary glaucoma, and exfoliative glaucoma) into analysis, a diagnosis of OSA was present in a significantly greater proportion of patients presenting with an initial parafoveal scotoma versus a nasal step (9 versus 0%, resp.; *P* = 0.001) [12]. The authors concluded that OSA is one of several IOP independent vascular risk factors that may increase the risk of an initial parafoveal scotoma. We agree that OSA might play an important role in many types of glaucoma but not OAG only.

Very few studies evaluated if different treatment strategies would influence the risk of glaucoma in OSA. Here we first reported that OSA patients with surgery are less associated with glaucoma. Regarding the surgery method versus glaucoma risk, pharyngeal surgery (HR = 1.86) is higher than nasal surgery (HR = 1.14). As far as we know practice parameters for surgical treatment for OSA in adults were first published in 1996 by the American Academy of Sleep Medicine (AASM) [3]; and clinical guidelines on the evaluation, management, and long-term care of OSA in adults were recently published by the AASM [13–15]. These guidelines included surgical modification of the upper airway but were based largely on expert consensus and were not intended to reflect a systematic evidence-based analysis [13–15]. Our current results show that both pharyngeal and nasal surgery are effective in reducing glaucoma risk. Instead, CPAP treatment only could not reduce the risk of glaucoma in OSA (HR = 1.65, 95% CI = 1.09–2.49) though it was used in the majority among all treatments. In Kiekens et al. study [16], they reported that nocturnal IOP increases more and is paralleled by a decrease in ocular perfusion pressure (OPP) during CPAP therapy, which could be the potential risk factor for higher prevalence of glaucoma. In another study proposed by Kadyan et al. [3], they found the prevalence of glaucoma in their study was similar to normal population data (2%, *P* = 0.429). They speculated this might relate to CPAP use in the majority of the OSA patients [3]. Although our result supports the concept that CPAP management did not reduce the risk of glaucoma in OSA patients, it cannot determine whether CPAP use has a deleterious influence on the development or progression of glaucoma.

OSA is a common disease, affecting approximately 2% of women and 4% of men residing in western communities [17]. In one recent review article, the authors highlight the lack of data regarding the prevalence of OSA in Asians [17]. They reported that OSA prevalence ranged from 3.7% to 97.3%. Two studies from Taiwan were reviewed; and both two used the method of modified questionnaire via telephone interviewing [18, 19]. In Liu et al.'s study, they found the prevalence of snoring among males was higher than females (57% versus 37%, *P* < 0.001). In addition, adults aged 40–59 years were more likely to snore (*P* < 0.001) [3]. In another study proposed by Chuang et al. [19], they reported that 51.9% (95% CI 51.13%–52.67%) of Taiwanese snore and 2.6% (95% CI 2.1%–3.1%) have witnessed apnea. Although the findings in the above two studies are interesting, it is difficult to directly compare our current result to those two because of different study design and sample size. The definition of OSA in the two studies was not based on PSG exam but on symptomatology.

Important findings were obtained from our current result. Younger patients with OSA had greater hazard ratios than older patients with OSA in glaucoma risk than non-OSA comparison cohort. Woman with OSA had greater hazard ratios than men with OSA in glaucoma risk than non-OSA comparison cohort. Similar finding was observed in another recent study by Lin et al. [4]. They reported that the adjusted hazard of OAG diagnosis among females during the 5-year follow-up period was 1.55 (95% CI, 1.04–2.31) times greater for those with OSA than for those without. However, among males, the adjusted HR for OAG diagnosis within the 5-year period for subjects with OSA was only 1.45 (95% CI, 1.02–2.16) than that of comparison subjects. Contrary to the general thought that older age and male patients should have higher risk of OSA or glaucoma risk, the clinical implication of this finding is to remind the clinicians of the higher risk of glaucoma in younger patients and female patients when being with OSA disease.

We believe our work has some strength. First, the strength of database was a large one with good sample randomization. Second, this dataset captures data on a broad range of subjects of different sociodemographic profiles unlike some smaller studies that recruit patients from a specific region which might not represent the whole population because of an overrepresentation or underrepresentation of individuals with certain sociodemographic characteristics in that area [9]. However, the current study also has some limitations. First, although we defined glaucoma totally relying upon claims data (ICD-9 coding from clinicians), which may be less accurate than diagnoses carried out individually through a standardized procedure. Second, selection bias did exist in this study. Because the NHI database only included patients who seek for treatment, for those who did not seek for help might have the chance to be recruited in the comparison cohort [4]. Third, we could not determine the severity of OSA from our database so that we could not directly evaluate the association between glaucoma and OSA severity. Finally, we could not understand the relationship between treatment strategy and OSA severity. Although surgery is better for OSA treatment with lower glaucoma risk, we need to be cautious in managing each case, especially considering the complications. In one recent study [20], the authors reviewed the safety of multilevel surgery in 487 consecutive patients with OSA and 1698 surgical procedures from January 2007 to May 2010. The overall complication rate was 7.1%.

### 4.2. Conclusion. 

OSA is associated with an increased risk of glaucoma in Taiwanese population. However, OSA patients having surgery are less associated with glaucoma and are more associated with glaucoma in female and younger age. Further research is warranted to understand why different OSA treatments may have variable risk for glaucoma.

## Figures and Tables

**Table 1:** The results of comparing baseline demographic status and comorbidity between comparison cohort and sleep apnea syndrome (OSA) cohort.

Variable	Comparison cohort	OSA cohort	*P* value
	*N* = 10112 (%)	*N* = 2528 (%)	
Age, years (SD)∗	45.1 (14.9)	45.1 (14.8)	0.9233
Follow-up, years (SD)			
Without glaucoma	4.4 (2.6)	4.5 (2.5)	<0.0001
With glaucoma	3.4 (2.6)	3.0 (2.5)	
Sex			>0.99
Female	2244 (22.2)	561 (22.2)	
Male	7868 (77.8)	1967 (77.8)	
Management for OSA			
Without treatment	—	1431 (56.6)	
With surgery only	—	296 (11.7)	
With CPAP only	—	617 (24.4)	
OSA with multiple modality	—	184 (7.3)	
Comorbidity			
Without any comorbidity	7273 (71.9)	1265 (50.0)	<0.0001
Hypertension	1975 (19.5)	902 (35.7)	<0.0001
Diabetes	764 (7.6)	264 (10.4)	<0.0001
CAD	830 (8.2)	498 (19.7)	<0.0001
Hyperlipidemia	1433 (14.2)	755 (29.9)	<0.0001

*t-test.

**Table 2:** Incidence rates of glaucoma and multiple Cox regression model measured hazard rates ratio between two study cohorts.

Variable	Comparison cohort			OSA cohort			Crude HR (95% CI)	Adjusted HR (95% CI)
	Event	PYs	Rate	Event	PYs	Rate		
Total	191	44535	42.89	101	11109	90.91	2.12 (1.67–2.70)	1.88 (1.46–2.42)
Age group								
0–44	42	22434	18.72	29	5576	52.01	2.77 (1.73–4.45)	2.42 (1.48–3.96)
45–54	54	11980	45.08	26	3019	86.12	1.90 (1.19–3.03)	1.64 (1.00–2.68)
≧55	95	10121	93.86	46	2514	182.95	1.95 (1.37–2.78)	1.65 (1.14–2.40)
Sex								
Female	44	9350	47.06	30	2309	129.94	2.75 (1.73–4.38)	2.23 (1.37–3.65)
Male	147	35185	41.78	71	8801	80.68	1.93 (1.45–2.56)	1.75 (1.30–2.35)
Comorbidity								
Without any comorbidity∗	84	32703	25.69	30	5671	52.9	2.06 (1.36–3.13)	2.40 (1.58–3.66)
Hypertension								
No	106	36464	29.07	51	7235	70.49	2.43 (1.74–3.39)	2.48 (1.76–3.50)
Yes	85	8071	105.31	50	3875	129.05	1.22 (0.86–1.73)	1.37 (0.96–1.97)
Diabetes								
No	157	41504	37.83	84	10022	83.82	2.22 (1.70–2.89)	1.99 (1.50–2.62)
Yes	34	3031	112.17	17	1087	156.32	1.34 (0.75–2.41)	1.29 (0.70–2.38)
CAD								
No	159	41148	38.64	68	8962	75.87	1.96 (1.48–2.61)	1.96 (1.46–2.62)
Yes	32	3387	94.47	33	2147	153.7	1.60 (0.98–2.60)	1.66 (1.01–2.72)
Hyperlipidemia								
No	140	38724	36.15	60	7944	75.53	2.09 (1.54–2.83)	1.95 (1.42–2.66)
Yes	51	5811	87.77	41	3166	129.51	1.46 (0.97–2.21)	1.60 (1.05–2.43)

Model adjusted for age, sex, hypertension, diabetes, CAD, and hyperlipidemia.

∗Model adjusted for age and sex.

PYs: person-years; rate: incidence rate, per 10,000 person-years.

**Table 3:** Glaucoma incidence rates and multiple Cox regression model measured hazard rates ratio among comparison cohort and OSA cohort with different managements.

Variable	Event	PYs	Rate	Crude HR (95% CI)	Adjusted HR (95% CI)
Comparison cohort	191	44535	42.89	ref	ref
OSA without treatment	62	6018	103.02	2.41 (1.81–3.21)	2.15 (1.60–2.88)
OSA with surgery only	7	1407	49.75	1.15 (0.54–2.45)	1.53 (0.72–3.29)
With pharyngeal surgery	5	858	58.25	1.34 (0.55–3.27)	1.86 (0.76–4.56)
With nasal surgery	2	549	36.45	0.84 (0.21–3.38)	1.14 (0.28–4.62)
OSA with CPAP only	27	2712	99.55	2.34 (1.56–3.50)	1.65 (1.09–2.49)
OSA with multiple modality	5	972	51.46	1.16 (0.48–2.82)	1.25 (0.51–3.04)

Model adjusted for age, sex, hypertension, diabetes, CAD, and hyperlipidemia.

PYs: person-years; rate: incidence rate, per 10,000 person-years.
